# COVID-19 and orthodontic treatment: current perspectives

**DOI:** 10.1590/2177-6709.26.3.e21ins3

**Published:** 2021-06-25

**Authors:** Nilesh MOTE, Shubhangi MANI, Kunal PALLAN, Rishikesh RATHOD

**Affiliations:** 1 Pravara Institute of Medical Sciences (Ahmednagar/MH, India).

**Keywords:** COVID-19, Orthodontic management, Vaccination

## Abstract

**Introduction:**

A pandemic was declared by the World Health Organization on 30th January 2020, the coronavirus disease (COVID-19) emerged, and led to standstill of Dentistry and Orthodontics.

**Description:**

The COVID-19 is a very multivariant disease. It affects in many different ways; the most reported symptoms resemble very much to that of a seasonal flu. Patients feel rising fever, dry cough and shortness of breath. There are two ways to handle them, the first being remotely guiding and helping them with aid of telecommunication, and second you can prepare the clinic by following all sanitization protocols and keep the clinic open only for such patients. Usage of Environment Protection agents, N95 masks, PPE kits and HEPA filters are some of the basic things to go about.

**Conclusion:**

With the non-stop change of scenario of the COVID-19, meticulous monitoring of the local situation and one eye on the latest instructions given by the WHO and Health ministry should be followed.

## RESUMO

**Introdução:**

Uma pandemia foi declarada pela Organização Mundial de Saúde em 30 de janeiro de 2020. A doença do Coronavírus (COVID-19) surgiu, e levou à paralisação da Odontologia e da Ortodontia.

**Descrição:**

A COVID-19 é uma doença muito multivariante. Afeta de muitas formas diferentes; e seus sintomas mais relatados assemelham-se muito aos de uma gripe sazonal. Os doentes sentem febre crescente, tosse seca e falta de ar. Há duas maneiras de lidar com eles, sendo o primeiro guiar e ajudar os pacientes remotamente com a ajuda das telecomunicações; e segundo, preparar a clínica, seguindo todos os protocolos de sanitização e manter a clínica aberta apenas para esses pacientes. A utilização de agentes de proteção ambiental, máscaras N95, kits EPI e filtros HEPA são algumas das coisas básicas a serem feitas.

**Conclusão:**

Com a mudança contínua do cenário da COVID-19, deve-se seguir um acompanhamento meticuloso da situação local, observando as últimas instruções dadas pela OMS e pelo Ministério da Saúde.

## INTRODUCTION

In these testing times of a global pandemic caused due to severe acute respiratory syndrome coronavirus 2 (SARS-CoV-2) infection, the practice of dentistry and orthodontics has come to a standstill. The coronavirus disease (COVID-19) was declared as a pandemic by the World Health Organization on 30th January 2020. In this situation, all governing and professional bodies have advised to handle only dental patients in emergency situations, also taking a great amount of precaution and preparation.

## BACKGROUND

The coronavirus disease (COVID-19) is believed to have originated from the city of Wuhan, in the Hubei province of China, and is caused due to infection by the now-called severe acute respiratory syndrome coronavirus 2 (SARS-CoV-2). The SARS-CoV-2 virus is a single-stranded RNA virus that belongs to a family called *Coronaviridae*, which includes the known Severe Acute Respiratory Syndrome coronavirus (SARS-CoV) of 2002, and even the Middle East respiratory syndrome coronavirus (MERS-CoV) that was seen in 2012.^1^ ^,^ ^2^ ^,3^

The virus genome has been sequenced and it was found to share 79.5% of the genomic sequence with the SARS-CoV.

## SOURCE OF TRANSMISSION AND SYMPTOMATOLOGY

On average, it takes 5 to 6 days for symptoms to develop from the time a person is exposed to the virus. Occasionally, it can take up to 14 days and rarely, even longer^1^ ^,^ ^4^ ^,5^.

The COVID-19 affects different people in different ways. The most common symptoms resemble very much to that of seasonal flu. Patients experience dry cough, rising fever and tiredness or shortness of breath. Some patients have even been documented having joint pains, headache, loss of taste or smell, sore throat, rashes and/or diarrhea. On the other side, there are asymptomatic patients who can act as “carriers” and act as a pool of infection.

Chest radiography study reveal ground-glass opacities in patients with advanced infections. Most of the patients with good immunity and no comorbidities brush away these symptoms in course of time, by developing the necessary antibodies. Others who have a compromised state develop complications like Severe Respiratory Distress situation or Pneumonia, and the downward spiral into the disease begins^2^ ^,^ ^6^.

SARS-CoV-2 binds to human angiotensin-converting enzyme 2 receptors. The salivary glands of humans are rich in these receptors and thus there is a high potential for transmission of COVID-19 via respiratory droplets in the air, which spread easily to a radius of no further than 6 feet, and then can enter the body directly through eyes, nose, ears and mouth, this would later infect the clinician, who may get critical or, worse, can become a carrier. 

Faces have also shown a certain titer level of this virus. An average person touches his face 23 times an hour, and then some of the other inanimate objects around them. Spread of virus can also happen in this way, as the virus can survive for up to three days on such objects and surfaces. Incubation period is known to last for one to two weeks^7^.

## CLINICAL PRACTICE OF ORTHODONTICS DURING COVID-19 PANDEMIC

Facing such a difficult and highly contagious disease, the government also recommends to handle only emergency patients or patients that will require minimal intervention.

There are two ways in which you can handle them:

1. Remotely guiding and helping them, with help of telecommunication.
2. You can prepare your clinic by following all sanitization protocols and then keep the clinic open only for such patients.

## REMOTE ASSISTANCE

Remote handling of the patient requires the orthodontist to have patience and understanding of the patient’s psyche. Conversation content should be well planned and thought of. Patients are not aware of the dental terminology, so more colloquial terms should be used. Much more of a listening attitude should be inculcated while they convey about their troubles. Use of calm tone and positive words is most important while the government-mandated restrictions are on. More than the orthodontist, it’s the patient who is suffering, as their appliance is still there and treatment will surely be prolonged. Since all this is being done on virtual platforms, encrypted end to end applications should be used, so that the patient also feels secure while this is being done, and the data should also be stored by the clinician. Orthodontic treatment requires meticulous and pragmatic approach from the clinician’s aspect, so that proper improvisations can be done for the treatment. Lastly, all these new protocols have to be done after confirmation from the Regional Dental associations, since these things rely on the interpretation of information given by the doctor and are also confidential.

Orthodontists should follow Alexander’s principle of “Let It Cook” more religiously, now that the wire sequence duration is prolonged, instead of getting anxious.

- Since most of the patients are in the adolescent category, with the school and colleges closed, unvaried calls should be made regarding the food types to be avoided to prevent breakages. Patients in the adult category will obviously be more understanding and will not be disobedient regarding the instructions.
- Regarding bonded molar tubes or brackets, patients can be asked to use a toothpick to get the loose appliances out, following instructions provided via video call.
- Use of elastics can be taught to the patient by either sending an on-line video or explaining them with help of a model, via a video call.
- With the public transport closed, an impinging wire can easily be cut with help of a nail cutter, cleaned with an alcohol-based sanitizer or by boiling it in water at 100ºC for 20 minutes. This can be done if the archwire is of lower gauge. In case of rigid rectangular wire, a heavy-duty cutter will be needed, which can easily be ordered on-line. Guiding the patient through a video is suggested at all times. Patient should be informed to hold the distal end of the wire secure, so as to prevent the risk of swallowing. Alternatively, a ball of wet cotton can provide temporary relief from the end of a poking wire, in case the patient has run out of relief wax.
- Regarding the expansion screws, patients can be reminded about it and instructed again, with help of a phone call. The expansion should be monitored via video call or by assessing photos of teeth, sent by the patient, and the expansion discontinued at the right time. In such situations, it is safer to err on the side of caution, and avoid over-expansion. Internet links on guidance for the patient to take dental photos should be sent to them in advance.
- Consideration should be given to terminate the procedures that are likely to cause problems if there is another wave of COVID-19, such as:
  1. Activation of slow/rapid expansion screw.
  2. Use of reverse pull headgear or Class III elastics.
  3. Elastics used for correction of crossbite/scissor bite/open bite/midline correction.
  4. Removable posterior bite-blocks to intrude the molars.
- Patients using removable functional appliances should be reminded to wear it, and also instructed regarding cleaning it. All patients, in general, should receive video explaining the hand hygiene protocol.
- In case of aphthous or traumatic ulcers, routine topical anesthetic ointments, which are available over the counter, should be recommended. A photographic examination should be performed, with regard to the size and severity of the ulcer, keeping in mind differential diagnosis of ulcers.
- Retention plates can easily be delivered with contactless delivery technique, and hoping it fits well. Use of the plate and instructions can, again, be done remotely.
- Since the use of ultrasonic scalers will be next to none, use of mouthrinse and a minimum of twice daily brushing protocol has to be more strictly followed and reminded multiple times.
- In case of aligner patients, their next aligner can be easily delivered without contact, and patients’ treatment progresses unhindered. If they feel their current aligner is incorrectly adjusted or any other issue, they can be asked to wear the previous aligner. Again, monitoring via photos taken by the patient is helpful, and the internet links for guidance to take correct dental photos should be sent to the patient in advance.
- In case patient does experience serious pain, 500-mg Paracetamol tablets can be prescripted.^8^ ^,^ ^9^

Once a list of urgent attention-needing cases is made, they can be called to the clinic after conducting a session on the phone, asking the necessary questions to establish a baseline regarding the condition of the patient. Any risk factor for COVID-19 disease and virus exposure history (contact history) has to be ruled out in the telephonic conversation.

## IN-CLINIC HANDLING

Before the patient visits the clinic, the following measures should be undertaken:

1. The clinic should be sanitized with Environmental Protection Agent (EPA).^10^ Magazine stand, brochures, displays and other unnecessary items should be removed, and only flat surfaces, which can be easily cleaned, should be present in the waiting room and operatory.^11^
2. The waiting room should be marked so that patients know where to sit, maintaining enough distance between the patients. Inform patient to obey the appointment time, so that proper sterilization and disinfection can be done in the meantime. A minimum number of patients should be in the waiting room, and all measures should be executed to decrease or eliminate the waiting time of a patient. A better way would be to ask the patient to sit in their car and wait for their turn. Hand hygiene instructions should be displayed in the waiting room along with an alcohol-based sanitizer, for the patients to use.^11^
3. The door knobs and door glasses should be touched only by people working in the clinic. Since the virus survives on inanimate objects and surfaces, this caution should be taken to prevent patients unwarranted contact with such surfaces.
4. Assistants and clinic personnel at desks should be instructed for these new techniques, and should be given the personal protection equipment (PPE) that is approved and adheres to all guidelines. A brief period of training of all staff is mandatory before resuming any clinical services.

After wearing the PPE kit, it can get really hot inside for the dentist and clinic personnel; so, the windows should be kept open, to allow continuous air exchange. As the virus is known to survive in surroundings for many hours, it would be advantageous to maintain a regular flow of fresh air. High Efficiency Particulate Air (HEPA) filters are mechanical air filters that work by forcing air through a fine mesh that traps harmful particles, such as pollen, pet dander, dust mites, and tobacco smoke. To be termed as HEPA grade, it must filter at least 99.97% of all particles with size between 0.15 and 0.2 µm, which is the Achilles heel of HEPA filters. So, when buying, if HEPA is 99.97% efficient then it can be used. But before entering into a panic buying situation, its better to be aware of products that are labeled as true HEPA, HEPA-like or HEPA type, and its performance should be certified by International Organization for Standardization (ISO 29463).^12^

5. Before starting all this, a thought should be given regarding the waste disposal system. Timely collection of biomedical waste should be ensured.

As soon as a patient enters the clinic, their temperature should be checked with a contactless infrared thermometer, since It’s been more than a year after the first case of COVID-19, and the disease has entered the Community Spread Stage, so history of travel does not hold much significance; but the patient can be asked about any history of basic seasonal flu symptoms and a history of contact with any known COVID-19 positive patients. Footwear needs to be kept outside clinic premises, and a protective foot-covering should be given to the patient, for covering the whole foot. Patients should be given a head cover, hand gloves and mask before they enter the operating area. It has been documented that SARS-COV-2 can’t survive in an oxidative environment, so preprocedural mouthrinse with 0.2% povidone iodine or 1% hydrogen peroxide should be done. Chlorhexidine mouthwash at 0.12% concentration does keep the viral load low to certain extent only for short period of time.^12^ ^,^ ^13^ It would be really helpful if a generalized set of rules information can be sent via messaging services, so that it can be kept in mind by the patient and, before they enter the clinic, they are well versed in all the precautionary measures.^5,^ ^14^ ^,^ ^15^ ^,^ ^16^

Various techniques to decrease chairtime, interval between appointments and overall treatment duration can be thought of, as follows:

1. A shift from fixed appliances to a self-ligating bracket system should be thought of, as there is reduced number of wires changes, the interval between appointments is minimum of six weeks, and with the better bracket manufacturing technique the rate of breakages may also be smaller.^8^
2. Treatment planning should be well chosen, and appliances or techniques that deliver maximum results with minimum chairside activation should be employed.
3. Attempts should be made to decrease the patient visits during the retraction process.
4. A shift to 0.018-in slot can be thought of in mild cases that do not require a lot of control, as the wire sequence to reach a 0.016 x 0.022-in stainless steel working wire is shorter.
5. Clear Aligner Technology: Boon for current times!?
  - Aligner technology can employ a digital scan instead of impressions, thereby decreasing chairtime and reducing contact with patient saliva.
  - To further reduce patient contact, digital treatment plans can be made without use of attachments, so that it reduces patient chairtime. For difficult movements where attachments are imperative, the placement can be delayed by a few weeks or months, to reduce current risk of exposure.
  - Space gaining in aligners can be achieved mostly via expansion and interproximal reduction (IPR). IPR should be done in the most non invasive way.
  - Aligners can be delivered to a patient with contactless delivery.
  - Instructions and maintenance protocol videos or internet links can be sent to the patient.
  - Regular remote monitoring via video calls and photos is possible. Chairside monitoring can be performed once in three months or only when non-tracking of teeth is detected.
  - If the teeth have moved correctly and treatment has tracked well, even the retainers can be fabricated and delivered remotely, from the last planned position of teeth, via digital treatment plan, without patient contact at all.

Various changes to be made by orthodontist:

1. Clinics should be designated with proper donning and doffing areas for PPE. Doctors should be wearing a full PPE kit, even if any aerosol emission procedure is not carried out. According to AAO (American Association of Orthodontics), Level 3 surgical masks are sufficient to protect against aerosolized coronavirus. According to recent CDC guidelines, N95 Respirator, provides greater protection.^17^ Minimal use of a high-speed air rotor handpiece should be adopted in routine practice from now on, as it generates aerosols that may contain the infective virus droplets from the asymptomatic patients’ saliva. Use of such handpiece is normally for removal of residual composite or enameloplasty. Shift to contra-angle handpiece should be done, with use of a syringe to drip water on the cutting area, in order to minimize aerosol generation.
2. With the handpiece out of question, only interproximal strips should be used for IPR procedures.
3. Technique of welding and soldering should be taught to assistants.
4. Multiple sets of basic armamentaria must be made, so that the time lapse between each patient can be minimized. For all patient, we should use all autoclaved instruments.
5. Handling and giving the elastics, NiTi coil springs, elastomeric chains and other auxiliaries can be performed by the assistant, to prevent contamination as much as possible.
6. Impression procedure should be bare minimum and if done, the impression should be disinfected with 2% Glutaraldehyde.
7. Fresh bondings and start-ups should be delayed for now, as the chairside time is to be minimized.
8. LED or UV curing light guns should be sanitized after every use.
9. Use of high strength composite as bite blocks can be thought of, so that they can be easily removed when not needed.

## VACCINATION AND IMPLICATIONS

A two-dose regimen of BNT162b2 vaccine conferred 95% protection against Covid-19 in persons 16 years of age or older. Safety over a median of two months was similar to that of other viral vaccines.^18^ Although the vaccine has been proven effective, questions have been raised about the longevity of the formed antibodies, since in orthodontic treatment, the treatment duration is longer than a year. So a patient who was vaccinated before, may not have antibodies by the end of treatment. Thus, safety measures need to be followed all time.

## CONCLUSION

The field of Orthodontics is ever-changing. There is a wide range of parameters and variables, all combined together in many permutations and combinations. Therefore, we are used to continuous evolution of the subject and practice. Change is the only permanent state, so we should face the current crisis with utmost precaution and attention. Treatment modalities will change, patient expectations will change, COVID-19 is an uninvited guest, but it will not leave soon, because the disease is new, its study is still not complete, and the situation is changing day by day. Therefore, innovative thinking and small tweaks will go a long way to help us. Health of our staff and our helping associates should not be compromised at any cost. Difficult times also call for difficult measures, and those should be taken keeping in mind the cost/risk-benefit ratio for the patient and the doctor. Current approach should be to minimize direct patient contact and provide maximum remote assistance and management. 
